# The Development of Risperidone-Loaded Microfibers via Centrifugal Spinning to Enhance the Palatability of a Potential Drug for Autistic Children

**DOI:** 10.3390/pharmaceutics17111403

**Published:** 2025-10-30

**Authors:** Sarah H. Alqahtani, Alhassan H. Aodah, Yasser A. Alshawakir, Bayan Y. Alshehri, Ali A. Alamer, Haya A. Alfassam, Fahad A. Almughem, Abdullah A. Alshehri, Essam A. Tawfik

**Affiliations:** 1 Advanced Diagnostics and Therapeutics Institute, Health Sector, King Abdulaziz City for Science and Technology (KACST), Riyadh 11442, Saudi Arabia; shalqahtani@kacst.gov.sa (S.H.A.); aaodah@kacst.gov.sa (A.H.A.); balshehri@kacst.gov.sa (B.Y.A.); aaalamer@kacst.gov.sa (A.A.A.); halfassam@kacst.gov.sa (H.A.A.); falmughem@kacst.gov.sa (F.A.A.); abdualshehri@kacst.gov.sa (A.A.A.); 2 Department of Experimental Surgery and Animal Laboratory, College of Medicine, King Saud University, Riyadh 12372, Saudi Arabia; yalshawakir@ksu.edu.sa

**Keywords:** autism spectrum disorder, risperidone, microfibers, centrifugal spinning, pediatrics

## Abstract

**Background/Objectives:** Children with autism spectrum disorder (ASD) frequently experience poor compliance with oral medication due to bitterness, unpleasant taste, and unsuitable dosage forms such as large tablets or capsules. Risperidone, a widely prescribed antipsychotic for managing ASD symptoms, is particularly challenging in this regard. The present study aimed to develop a novel sucrose-based microfiber drug delivery system to improve the palatability, acceptance, and bioavailability of risperidone in pediatric patients with ASD. **Methods:** Risperidone was incorporated into sucrose microfibers using centrifugal spinning technology. Fiber morphology was characterized by scanning electron microscopy (SEM). Drug loading (DL), encapsulation efficiency (EE%), and disintegration time were measured. In vitro drug release and cytotoxicity assays were performed using human foreskin fibroblast cells (HFF-1). An in vivo palatability and preference study was conducted in male BALB/c mice to evaluate the acceptability of the formulation compared with a commercial risperidone oral solution. **Results:** SEM analysis revealed smooth, bead-free, non-porous fibers with uniform morphology and size distribution. The formulation showed a rapid disintegration time of ~3 s, DL of 30 ± 5 µg/mg, and EE% of 60 ± 10%. Approximately 50% of risperidone was released within 15 min. Cytotoxicity testing confirmed that concentrations ≤ 125 µg/mL maintained high cell metabolic activity, indicating biocompatibility. In vivo, the microfiber solution demonstrated a strong preference (93%) compared with the commercial oral solution (30%). **Conclusions:** Risperidone-loaded sucrose microfibers represent a promising fast-dissolving oral delivery system for children with ASD. This child-friendly formulation improves palatability and compliance while maintaining safety and drug release performance.

## 1. Introduction

Autism spectrum disorder (ASD) is a neurodevelopmental disorder that impacts brain function and is marked by varying degrees of impairment in social interaction, communication, and behavior. According to the World Health Organization (WHO), ASD affects approximately one in every 100 children globally [1], while globally, it was estimated in 2021 that approximately 61.8 million individuals were on the autism spectrum [2]. Many studies have investigated the link between autism and feeding issues, and revealed that a significant number of individuals, most of whom are children, experience difficulty swallowing pills [2,3]. It has been reported that approximately 90% of autistic children struggle to take any oral substances, including food and medicines [4]. A major characteristic of children with ASD is their food selectivity, commonly known as picky eating, which has been documented in the history of ASD research [5,6,7]. The refusal to take any oral supplements, such as medications, whether in solid or liquid form, can lead to serious consequences, including delayed treatment, nutritional deficiencies, and other related health issues [8]. The main reasons for resistance to medication among children with ASD are often due to the bitter taste or the large size of tablets or capsules, making them challenging to swallow or chew [9]. However, it is worth noting that some children with ASD also suffer from dysphagia [10]. Although there are limited data available to establish a strong connection between ASD and dysphagia. A lot of efforts have been made to help children with ASD overcome difficulties in pill-swallowing, including behavioral training, which has shown partial progress in some cases [11]. There is currently no medication that can completely cure autism [12]; however, several antipsychotic drugs have been found to help manage or reduce its associated symptoms, for instance, risperidone, aripiprazole, fluoxetine, and other pharmacologic agents [13].

**Figure 1:**
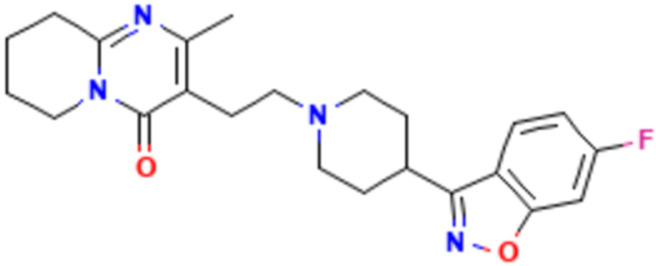
The chemical structure of risperidone was obtained from PubChem https://pubchem.ncbi.nlm.nih.gov/compound/Risperidone#section=Structures (accessed on 23 September 2025).

Risperidone is a second-generation (atypical) antipsychotic agent primarily prescribed for the treatment of schizophrenia. It exhibits poor aqueous solubility and, based on the Biopharmaceutics Classification System (BCS), is categorized as a Class II compound characterized by low solubility in water-based environments [14]. It is commonly used to reduce schizophrenia symptoms that involve aggressive behavior, self-harm, and extreme mood swings [15]. The chemical structure of risperidone is shown below (Figure 1):

Risperidone received Food and Drug Administration (FDA) approval in 2006 to be prescribed for children with ASD 5 years of age and older, and the recommended dose for children is varied from 0.5 to 3.5 mg daily, depending on age and ASD severity [16]. While the exact mechanism of risperidone remains unclear, it is suggested that its therapeutic effects are primarily due to dopamine receptor inhibition [17]. The hyperactivity of dopamine is linked to different mental disorders and could affect the behavior of individuals [18]. Risperidone has been demonstrated to bind various dopamine receptors, including D2 dopamine receptors and HT2A/2C serotonin receptors, and therefore blocks the dopamine release [19]. There are different commercially available dosage forms of risperidone, such as tablets, solution, and long-acting injections, and the liquid dosage form is often more suitable for pediatric use in general and for children with ASD in particular [20,21]. Yet, parents complain about the resistance and rejection of the drug due to its strong, bitter taste [22]. Clinical therapists used exercises to help children with pill-swallowing by strengthening their gastrointestinal muscles. Unfortunately, these trials have not shown any significant improvements. Some parents report only minimal progress in drug acceptance, even after extended periods of training [23]. Recent efforts to improve risperidone formulations have focused on enhancing physicochemical stability and patient acceptability. A recent study developed medicated gummies containing coated risperidone pellets for autistic children with ADHD, demonstrating stable, controlled drug release and good palatability. Such child-friendly formulations represent promising strategies to improve treatment adherence in autism therapy [24].

The development of rapidly dissolving sugar-based drug-loaded fibers presents a promising solution to the challenges that ASD patients face while taking their medication. This approach involves incorporating risperidone into a sucrose-based microfiber using a centrifugal spinning technique. This method has attracted attention in the pharmaceutical industry due to its potential to reduce anxiety and discomfort during drug intake, thereby making treatment more acceptable for children with ASD. Additionally, the fibers are rapidly dissolved and offer an advantage for patients with physical or mental disabilities. Another key advantage of this approach is its ability to deliver bitter-tasting drugs in a palatable and cost-effective dosage form, compared to other techniques. While sucrose offers advantages in fiber formation and palatability, its high concentration may pose potential dental health risks, particularly in pediatric use. Therefore, the incorporation of sucrose in such formulations should be balanced with strategies to minimize dental exposure and ensure safe long-term use.

The primary goal of this research is to optimize risperidone administration for children with ASD by developing fast-dissolving sucrose-based microfibers loaded with risperidone, designed to enhance the drug’s taste and treatment adherence. The formulation was prepared using a centrifugal spinning technique. The characterization includes analysis of fiber diameter, drug loading, encapsulation efficiency, drug release profile, and disintegration time. Other evaluations were conducted, including both in vitro and in vivo studies.

## 2. Materials and Methods

### 2.1. Materials

Risperidone, polyvinylpyrrolidone (PVP, average molecular weight ≈ 1,300,000), sucrose (>99%), sodium phosphate dibasic (≥99.0%), potassium chloride (99.0–100.5%), sodium chloride (≥99.5%), and potassium phosphate monobasic (≥99.0%) were all obtained from Sigma-Aldrich (St. Louis, MO, USA). Dulbecco’s Modified Eagle Medium (DMEM) supplemented with 10% (*v*/*v*) fetal bovine serum (FBS), 100 U/mL penicillin, and 100 μg/mL streptomycin was also sourced from the same supplier. Analytical-grade methanol was purchased from Scharlab (Barcelona, Spain), and hydrochloric acid (36.5–38.0%) from BDH (Dubai, United Arab Emirates). The human foreskin fibroblast cell line (HFF-1; ATCC-SCRC-104) was obtained from the American Type Culture Collection (ATCC, Manassas, VA, USA). The MTS assay reagent (CellTiter 96^®^ Aqueous One Solution Cell Proliferation Assay) was provided by Promega (Southampton, UK). Risperidone oral solution (1 mg/mL) was purchased from a local pharmacy (Riyadh, Saudi Arabia).

### 2.2. Preparation of Centrifugal-Spun Microfibers

The drug-loaded fibers were fabricated by initially blending 95% (*w*/*w*) sucrose with 5% (*w*/*w*) risperidone in a mortar and pestle for 5 min to create a homogenous physical mixture and to obtain a uniform mixture before fiber formation, following the modified method described in a previously published study [25]. The centrifugal spinning device was preheated for 10 min. Five grams of the physical mixture (PM) was placed into a commercial centrifugal spinning apparatus (ET-MF01, 520 mm, manufactured in Guangzhou, China and purchased from a local market, in Riyadh, Saudi Arabia), equipped with a heating unit, a large stainless-steel circular pan (520 mm in diameter), and an aluminum spinneret featuring a concave cavity designed to hold the loaded materials and a slight gap between the two plates. The spinning head was pre-warmed to a temperature range of 150–180 °C, and the rotational speed was set between 2000 and 2400 RPM. When the maximum rotational speed was reached, the centrifugal force then released the material through the spinneret gap to form microfibers. Blank fibers were prepared by the same method, but with only sucrose.

### 2.3. Scanning Electron Microscope (SEM)

Both drug-loaded and blank fibers’ morphological characterization and diameter were evaluated by SEM (JSM-6010 PLUS/LA, JOEL Inc., Peabody, MA, USA). Fibers were observed at an accelerating voltage of 5 kV. The average of at least 60 measurements of the fiber size was recorded for the drug-loaded and blank fibers to provide the average size of the fibers.

### 2.4. Disintegration Test of the Blank and Drug-Loaded Microfibers

The disintegration time was evaluated using a modified method of the Petri dish assay, as previously described by Alsulami et al. [26]. Briefly, a square section 2 cm × 2 cm from both the blank and drug-loaded fibers was placed into separate Petri dishes filled with 8 mL of phosphate-buffered saline (PBS), pre-warmed to 37 °C, and adjusted to a pH of 6.8 to mimic the oral cavity for a nearly realistic assessment.

### 2.5. Microfiber Physicochemical Characterization Using X-Ray Diffraction (XRD) and Fourier Transform Infrared (FTIR)

To track the change in crystal form to amorphousness and observe characteristic functional groups, X-ray diffraction (XRD) and Fourier transform infrared (FTIR) techniques were used for sucrose, risperidone, and PM in a ratio of 95% sucrose and 5% risperidone (in powder form), as well as for blank and drug-loaded fibers. The FTIR spectra were recorded by a Nicolet iS20 FTIR Spectrometer (ThermoFisher Scientific, Waltham, MA, USA) in the range of 4000 cm^−1^ to 600 cm^−1^ with a spectral resolution of 4 cm^−1^. The MiniFlex XRD benchtop diffractometer from Rigaku (Tokyo, Japan) was used to evaluate the effects of the fiber formation process on the crystallinity of sucrose over a diffraction angle (2θ) ranging from 2° to 50°, at a scanning speed of 5°/min, a voltage of 40 kV, and a current of 15 mA, using Cu Kα radiation [27].

### 2.6. Determination and Quantification of Risperidone Using High-Performance Liquid Chromatography (HPLC)

The quantitative analysis of risperidone was performed using a Waters e2695 HPLC system equipped with a Waters 717 plus autosampler, a Waters 600 binary pump, and a Waters 2489 UV detector (Waters Technologies Corporation, Milford, MA, USA). Separation was achieved under isocratic conditions using a mobile phase composed of 0.1% triethanolamine (TEA) adjusted to pH 3.9 with formic acid, methanol, and acetonitrile in a ratio of 60:5:35 (*v*/*v*/*v*). Risperidone samples were dissolved in a 1:1 (*v*/*v*) mixture of methanol and phosphate-buffered saline (PBS; pH 6.8). The PBS was prepared by dissolving 1.6 g NaCl, 0.04 g KCl, 0.288 g Na_2_HPO_4_, and 0.049 g KH_2_PO_4_ in 200 mL of distilled water, with the pH adjusted to 6.8 using HCl. Chromatographic separation was carried out on an XBridge C18 column (5 µm, 4.6 × 250 mm), maintained at 25 °C. Risperidone exhibited a retention time (Rt) of approximately 7.2 min. The flow rate was set to 1 mL/min, with an injection volume of 10 µL, and UV detection was performed at 230 nm.

### 2.7. Determination of Drug Loading (DL) and Encapsulation Efficiency (EE%) of Risperidone Microfibers

To determine the DL and EE% of the risperidone-loaded fibers, 5 mg of each sample was dissolved in 6 mL of phosphate-buffered saline (PBS, pH 6.8) and maintained at room temperature for six hours to achieve complete dissolution. The resulting solutions were then analyzed using the previously described HPLC method. The DL and EE% were calculated using the following equations:

(1) $$DL\;=\;\frac{Entrapped\;drug\;amount}{wieght\;of\;the\;fibers}$$

(2) $$EE\;\%\;=\;\frac{Actual\;amount\;of\;drug}{Theoretical\;amount\;of\;drug}\;\times \;100$$

### 2.8. Drug Release Determination of Drug-Loaded Fibers

To determine the drug release, 20 mg pieces of drug-loaded fibers were added to vials containing 15 mL of PBS (pH 6.8) in a shaking incubator at 37 °C and 100 rpm, following non-sink conditions of [28]. 1 mL of each sample was withdrawn at 12 time points starting from 1 to 360 min, and fresh 1 mL of pre-warmed PBS (pH 6.8) was added back to the vials. The cumulative release (%) was calculated using the following equation [29]:

(3) $$Cumulative\;release\;\%\;=\;\frac{Cumulative\;drug\;amount}{Theoretical\;drug\;amount}\;\times \;100$$

### 2.9. In Vitro Cytotoxicity Evaluation Using MTS Assay

The in vitro cytotoxicity of risperidone was determined using the colorimetric MTS assay [3-(4,5-dimethylthiazol-2-yl)-5-(3-carboxymethoxyphenyl)-2-(4-sulfophenyl)-2H-tetrazolium], which measures formazan production resulting from the reduction in tetrazolium salts by metabolically active cells. Various concentrations of risperidone were tested on the metabolic activity of human foreskin fibroblast (HFF-1) cells following a modified protocol by Alamer et al. [30]. HFF-1 cells were cultured in complete DMEM until reaching approximately 80% confluence. Adherent cells were then detached using 0.25% trypsin, incubated for 5 min, harvested, and seeded in 96-well plates at a density of 1.5 × 10^4^ cells per well. The plates were incubated overnight at 37 °C in a humidified atmosphere with 5% CO_2_. After 24 h, 100 µL of risperidone solutions with increasing concentrations (0.7–1000 µg/mL) were added to the wells. Treatments were maintained for 24, 48, and 72 h. Cells exposed to 0.1% Triton X-100 served as the negative control, while cells incubated with DMEM only were used as the positive control. Following incubation, the medium was replaced with 100 μL of fresh DMEM, and 20 μL of MTS reagent was added to each well. The plates were further incubated for 3 h, and cell viability was quantified by measuring absorbance at 492 nm using a microplate reader (Cytation 3, BioTek Instruments Inc., Winooski, VT, USA). The percentage of cell viability was calculated using Equation (4) [31]:

(4) $$Cell\;Viability\;\%=\;\frac{(S\;-\;T)\;}{H\;-\;T\;}\;\times \;100\;$$

where *S* represents the absorbance of risperidone-treated HFF-1 cells, *T* is the absorbance of cells treated with Triton X-100, and *H* is the absorbance of cells incubated with DMEM.

### 2.10. In Vivo Assessment

#### 2.10.1. Animals and Housing

Fifteen adult male BALB/c mice weighing approximately 25 ± 1 g were obtained from King Saud University (Riyadh, Saudi Arabia) and individually housed in plastic tub cages equipped with water bottles. Mice were adapted to the experimental cages, which were prepared using identical bedding material and matched the size and color of their home cages. Each cage was equipped with two stainless steel-tipped bottles containing steel balls to prevent leakage. The animals underwent a week of training before the experiment. They were maintained under standard laboratory conditions, with a 12:12 h light–dark cycle at a temperature of 22 °C and a relative humidity of 55% RH. Throughout the study, mice had free access to a standard diet supplemented with multivitamins purchased from Mucedola (Settimo Milanese, Italy) and deionized water, except during specific periods of water restriction. The experimental protocol and the use of animals in this study were reviewed and approved by the Research Ethics Committee at King Saud University (approval number KSU-SE-24-36).

#### 2.10.2. Experimental Design: Two-Bottle Preferences Test

To evaluate the palatability of the drug formulation through a two-bottle preference test, animals were divided in 3 groups: the control group had access to water only in both bottles, treated group (1) had access to water in bottle A and Risperidone oral solution (1 mg/mL) in bottle B, and treated group (2) had access to water in bottle A and the drug-loaded fiber solution (1 mg/mL) in bottle B. each mice was offered a choice between two bottles, one containing deionized water and the other containing the drug solution. Bottle positions were randomly placed in each session to prevent side bias. Fluid intake was measured, and a preference ratio was calculated to determine the acceptance or rejection of the drug formulation. The drug-loaded microfiber was introduced, and the same testing procedures were repeated daily. The control group was housed separately and underwent the same protocol, with both bottles containing deionized water as a baseline for comparison.

Before the experiment, the mice underwent water restriction for 6 h before each training or testing session. The experiment was conducted in a ventilated cabinet (Bio-C36 Techniplast, Buguggiate, Italy) with the same temperature and humidity levels to match the housing environment, thereby eliminating any distressing factors. Food was removed during the 10 min experiment to ensure the animals had access only to the bottles, as food could be a distracting factor. To ensure proper functionality, the bottle tips were positioned evenly with respect to the cage floor. The mice were trained on a short access according to a modified method to drink at least 10–15 mL of water within 10 min to prepare them for the test [32]. To prevent any bias from side preferences, the bottle positions were switched once during the experiment. After each training and testing session, the mice were given free access to water and food.

#### 2.10.3. Behavioral Observations and Data Analysis

Consumption levels were recorded for each session, and statistical analyses were performed to evaluate differences in fluid intake between the drug solution and water. The preference score was calculated using the following equation:

(5) $$Preference\;score\;\%\;=\;\frac{Vd}{Vd\;+\;Vw}\;\times \;100$$

where *Vd* represents the volume of the drug solution consumed, and *Vw* represents the volume of water consumed. A preference score of ≥50% indicates that the drug formulation was preferred over water, while a score of <50% suggests a preference for water.

This study followed the modified version of the protocol described by Gaillard et al. [33] which adhered to ethical standards to minimize animal stress. No surgical interventions, pathogen exposure, or blood collection were involved.

### 2.11. Statistical Analysis

SEM image analysis was conducted using ImageJ software (Version 1.54p, National Institutes of Health, Bethesda, MD, USA). XRD and HPLC data were processed and visualized using OriginPro^®^ 2021 (OriginLab Corporation, Northampton, MA, USA). In vitro and in vivo experimental data were analyzed using Microsoft Excel (2019). All results are expressed as the mean ± standard deviation (SD) based on a minimum of three independent measurements.

## 3. Results and Discussion

### 3.1. Morphological Characterization of the Fabricated Microfibers by Scanning Electron Microscopy (SEM)

**Figure 2:**
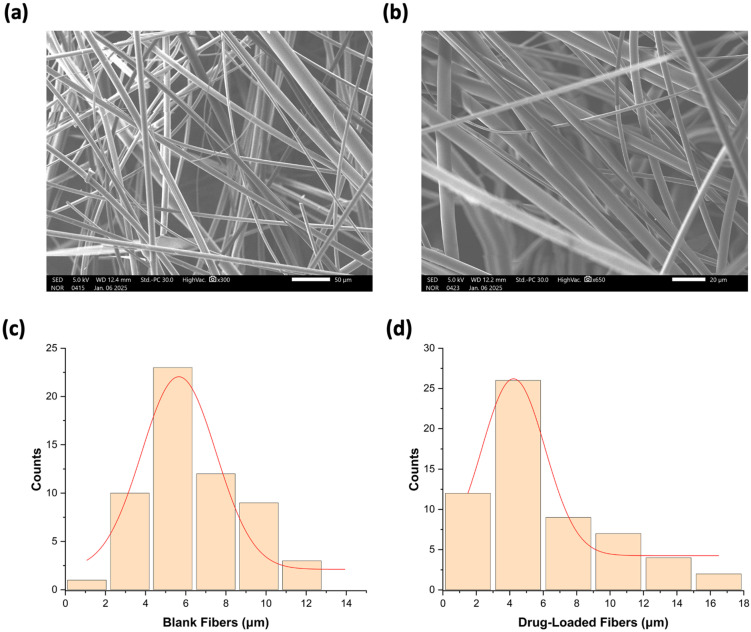
SEM image of (**a**) blank fibers and (**b**) drug-loaded fibers. The diameter distribution of (**c**) blank fibers was 6.8 ± 3 μm, and (**d**) drug-loaded fibers was 5.8 ± 3 μm.

The successful fabrication of the blank and drug-loaded fibers was examined using SEM, and the results are presented in Figure 2. SEM imaging revealed a smooth structure in both blank and drug-loaded fibers, with a non-porous, bead-free morphology. More importantly, no drug crystals were observed on the surface, suggesting an amorphous state, which is in agreement with the XRD results in Section 3.3. It also indicates that the drug is evenly distributed within the sucrose microfibers in alignment with previously reported studies [28,34]. The analysis also revealed that the average diameter of the drug-loaded fibers was 5.8 ± 3 μm, while the blank fibers had a slightly higher diameter of 6.8 ± 3 μm. The similarity in fiber diameters suggests consistency in the fabrication process between the blank and drug-loaded fibers, which is considered a critical aspect of drug delivery system development.

### 3.2. Disintegration Test of the Drug-Loaded Microfibers

**Figure 3:**
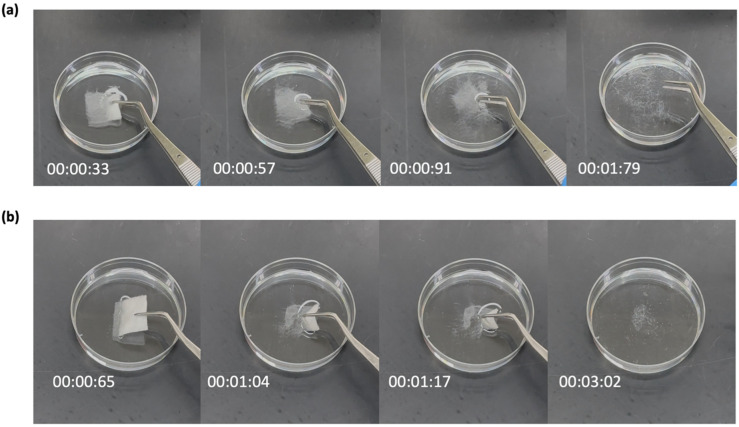
The disintegration profiles of (**a**) blank microfibers, and (**b**) risperidone-loaded microfibers in PBS with pH 6.8. Blank microfibers dissolved in around 2 s, while drug-loaded microfibers dissolved in around 3 s.

The assessment of disintegration is crucial for evaluating any oral dosage form formulated for use within the mouth cavity [35]. The U.S. Food and Drug Administration (USFDA) requires that the disintegration time for the oral disintegrating dosage forms should not exceed 30 s [36]. This timeframe represents a standard to ensure the dosage forms disintegrate rapidly in the oral cavity, which supports the drug absorption and improves patient compliance [37,38,39]. Both the blank and drug-loaded fibers quickly disintegrated when placed in Petri dishes containing PBS with a pH of 6.8. As shown in Figure 3, the blank fibers dissolved slightly faster at 2 s, while the drug-loaded fibers took approximately 3 s to disintegrate. This insignificant difference may reflect on the influence of the drug content on the fiber’s behavior once exposed to wet conditions [40]. Moreover, the presence of sucrose enhances the disintegration of fibers, as sucrose is known for its ability to absorb moisture from the surrounding environment, thereby facilitating the breakdown of the microfibers into smaller particles upon contact with saliva [41,42]. Consequently, this enhances the rapid disintegration of the drug-loaded microfibers.

### 3.3. X-Ray Diffraction (XRD) and Fourier Transform Infrared (FTIR)

The XRD and FTIR analyses were conducted to examine the physical characteristics of the raw materials, including sucrose, risperidone, their PM, as well as the blank and drug-loaded microfibers. The crystallinity and the spectrochemical structures of the pure sucrose and risperidone were described in Table 1.

**Figure 4:**
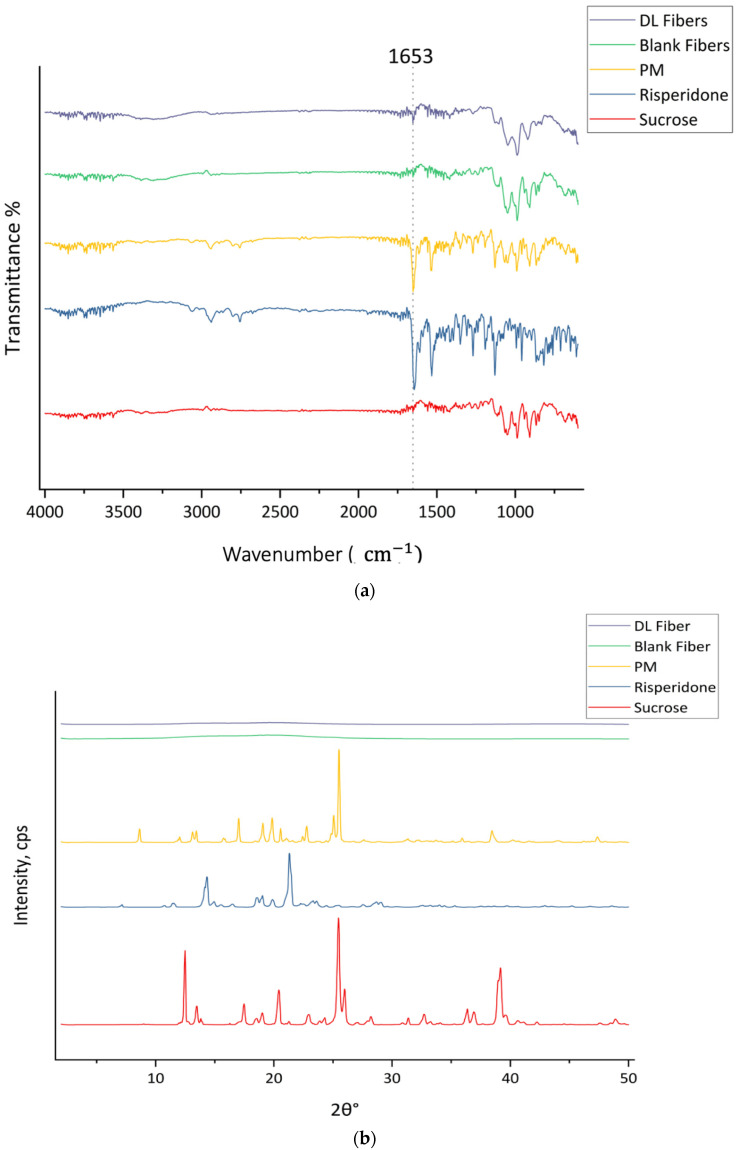
(**a**) FTIR transmission spectra and (**b**) XRD Bragg reflection patterns of sucrose, risperidone, and their PM, blank microfibers, and drug-loaded microfibers.

Figure 4 presents the FTIR and XRD profiles of pure sucrose and risperidone, their PM, and both blank and drug-loaded microfibers. The FTIR transmittance plot of the PM sample showed blend peaks that related to the risperidone and sucrose, especially the appearance of a strong peak at 1652 cm^−1^ for C=O stretching from risperidone and in addition to the peaks around 2940 and 2800 cm^−1^, which contribute to the possible C-H stretching vibration from both risperidone and sucrose, confirming that the materials are still in their original forms without significant interaction. The spectrum of the blank fibers exhibited the same characteristic peaks of sucrose, including O-H stretching between 3500 and 3000 cm^−1^, C-H stretching at 2943 and 2890 cm^−1^, and C-O stretching from the C-O-C linkage at 1116 cm^−1^, along with peaks in the fingerprint region corresponding to the C-C of carbohydrates. The spectra of drug-loaded fibers exhibited functional groups of both risperidone and sucrose, similar to those of the PM. Notably, the peak at 1653 cm^−1^ corresponds to the C=O stretching of the lactam in the risperidone structure, as labeled in Figure 4a. These results were aligned with those obtained in a previous study by Sbârcea et al., where the IR results showed the appearance of distinct spectra representing risperidone’s structure, particularly the carbonyl peak at 1648 cm^−1^ and the aryl fluoride peak at 1130 cm^−1^ [49].

The results are presented in Figure 4a. Furthermore, both sucrose and risperidone represent a crystalline structure in the XRD pattern, as shown in Figure 4b, with distinguished signals at 2θ° of 12.5°, 13.5°, 17.5°, 20.4°, 25.5°, 26°, 36.4°, and 39.2° for sucrose and at 2θ° of 14.3°, 18.6°, 19°, 19.9°, 21°, 23.3°, and 28.7° for risperidone. The PM represents sharp diffraction peaks at 2θ° of 8.7°, 12.1°, 13.1°, 13.5°, 17°, 19.1°, 19.9°, 20.6°, 22.8°, 25.1°, 25.5°, 38.5°, and 47.4° due to the phase separation of the pure materials before the centrifugal spinning process, which confirms that risperidone and sucrose remained in their crystalline form. In contrast, the lack of distinct Bragg reflections in the drug-loaded and blank fibers suggested that the crystalline forms of sucrose and risperidone were successfully converted to an amorphous form through molecular dispersion during the centrifugal spinning process without phase separation.

These findings are consistent with previously published work on the investigation of the lack of intense peaks for miconazole in the drug-loaded fibers after centrifugal spinning the sugar-based fibers, indicating the good molecular transformation of miconazole [25]. The centrifugal spinning of risperidone microfibers was consistent with the findings obtained from a recent study by Bessarabov et al. on the preparation of centrifugal fibers for nimesulide delivery [50]. In addition, another solid dispersion study developed a method for centrifugal spinning of sucrose-based microfibers loaded with olanzapine and piroxicam, which showed successful drug incorporation and complete conversion of drugs to the amorphous form [28]. The FTIR and XRD results obtained in this study align with previous studies, suggesting that the drug is well integrated into the sucrose fiber, which may promote drug release and stability.

### 3.4. Determination and Quantification of Risperidone

**Figure 5:**
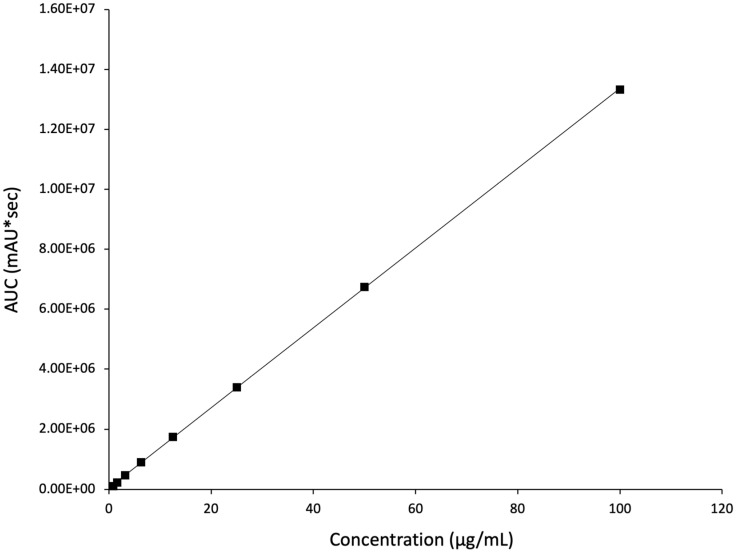
The developed HPLC calibration curve for risperidone demonstrates excellent linearity (R^2^ = 0.9999).

Risperidone quantification was performed using an HPLC method described in Section 2.7. A rapid separation of the drug happened within 7.2 min, i.e., retention time (Rt). The regression equation was determined as y = 133,045x + 55,376, with a coefficient of determination (R^2^) of 0.9999 (Figure 5). The calibration curve presented clear evidence of excellent linearity and the successful separation of the drug developed by this method.

### 3.5. Drug Loading (DL) and Entrapment Efficiency (EE%) of Risperidone-Loaded Microfibers

The DL and EE% of the microfiber loaded with the risperidone were found to be 30 ± 5 μg/mg and 60 ± 10%, respectively. These values were determined using the HPLC method developed in Section 2.7. The drug loading is influenced by several factors, such as the preparation method, formulation parameters, the physicochemical properties of both the polymer and the drug, and the interactions between the drug and the matrix [51]. The partial miscibility between risperidone, which has a hydrophobic nature, and the hydrophilic sucrose matrix, especially in the sucrose molten state (melting point ~186 °C), is likely due to thermodynamic incompatibility [52]. This may lead to phase separation, where risperidone particles aggregate and do not unify within the sucrose matrix, which limits the maximum actual drug loading. Similar studies have reported an EE% between 50% and 70% for sucrose-based fibers [25,53], suggesting that while sucrose can absorb moisture and increase disintegration time, it may also reduce compatibility with hydrophobic drugs like risperidone.

### 3.6. Drug Release Determination of Risperidone-Loaded Microfibers

**Figure 6:**
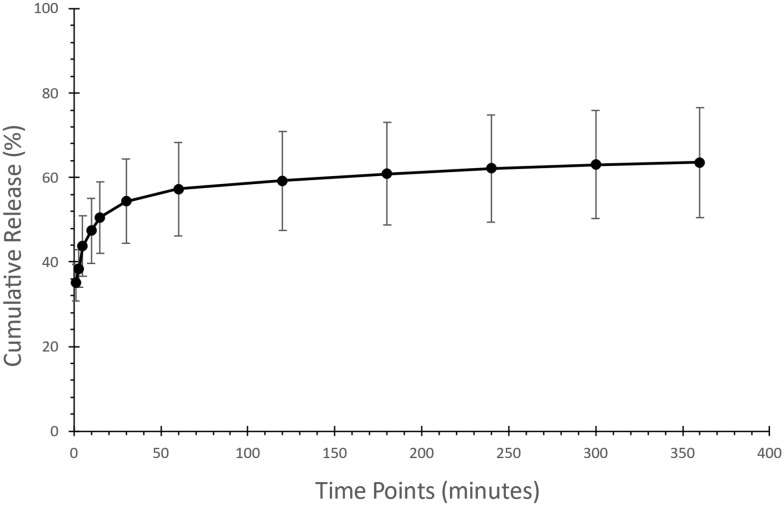
Assessment of drug release of the risperidone-loaded microfibers showed an initial 50% burst release within the first 15 min, followed by maximum drug release after 360 min.

The in vitro drug release determination is essential to provide a predictive assessment of a drug’s dissolution behavior and bioavailability to ensure consistency, efficacy, and quality control [54]. Sucrose microfibers loaded with risperidone were tested in PBS with a pH of 6.8. The initial release showed a rapid release of 50% within 15 min, as shown in Figure 6, followed by an increase to approximately 60% after 180 min. The release has reached a plateau at around 60% during the 360 min test, confirming the formulation’s maximum drug release. These results show reduced drug release compared to a previous study [25] that utilized sucrose-based fibers and centrifugal spinning to formulate BCS class II drugs, similar to risperidone.

Since the drug was released within 360 min, the dissolution rate may be influenced by the components of the microfiber, along with the drug-to-polymer ratio, which may affect the release of the drug [55] as well as the characteristics of the surrounding release medium [56]. Moreover, the amount of drug being loaded in the microfibers has a high impact on the amount of the drug being released from the matrix. When the drug loading is higher, it results in a rapid release, which is then followed by a slower and gradual release phase [57,58]. These results highlight the complex relationship between the components of any formulation and the manner in which the drug is released.

In addition, incomplete drug release can be linked to the low solubility of risperidone in water, as the dissolution medium used was a water-based salt solution -PBS- as mentioned previously, risperidone is classified as a BCS Class II drug, meaning it has low solubility but high permeability, as described [59]. Previous studies indicate that the plain drug release of risperidone in similar conditions does not exceed 30% [60,61], whereas our formulation reached 60% release after 360 min. The dissolution study confirmed that sucrose fibers loaded with risperidone effectively showed rapid drug release, which could enhance the palatability and adherence to risperidone medication. Further understanding of the limitations of having a maximum drug release at 60% is required.

### 3.7. In Vitro Cytotoxicity Assessment of Risperidone

**Figure 7:**
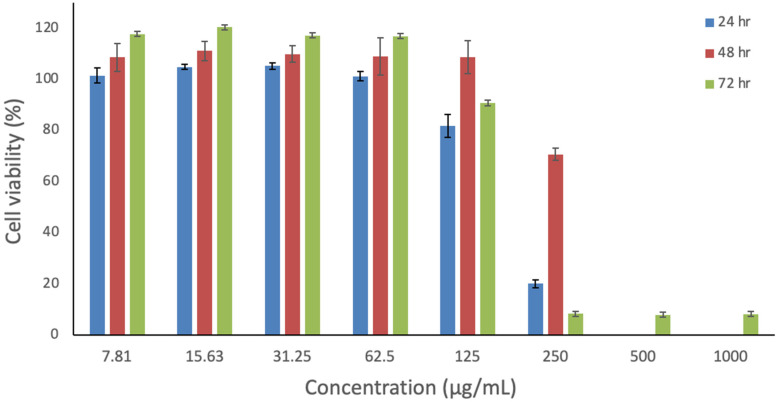
In vitro assessment of risperidone’s impact on HFF-1 cells over 24, 48, and 72 h. The data represent the cellular viability (%) and are presented as the average ± SD (n = 3).

The in vitro cytotoxicity of risperidone was evaluated to determine its safety profile. The MTS assay was employed to measure the effect of various risperidone concentrations on the metabolic activity of human dermal fibroblast cells (HFF-1), which are representative cells due to their high sensitivity. Cells were exposed to risperidone for 24, 48, and 72 h, as shown in Figure 7. Higher toxicity levels were observed at concentrations ≥ 250 µg/mL, where cell viability was below 20%. During the 48 h exposure, 0% cell viability was observed at the two highest concentrations, while cell viability reached 70% at 250 µg/mL after 48 h of exposure. Notably, across all three time points of exposure, a safe profile was evident at concentrations ≤ 125 µg/mL with cells exceeding 80% of viability. Risperidone demonstrated a lower level of toxicity in comparison to other atypical antipsychotic drugs, in agreement with the result of Dwyer et al. [62]. Overall, this study suggests that risperidone is safe for use at a concentration of ≤125 µg/mL, and further investigation on different cell lines is required to confirm this observation.

### 3.8. In Vivo Palatability Assessment: Two-Bottle Preferences Test

**Figure 8:**
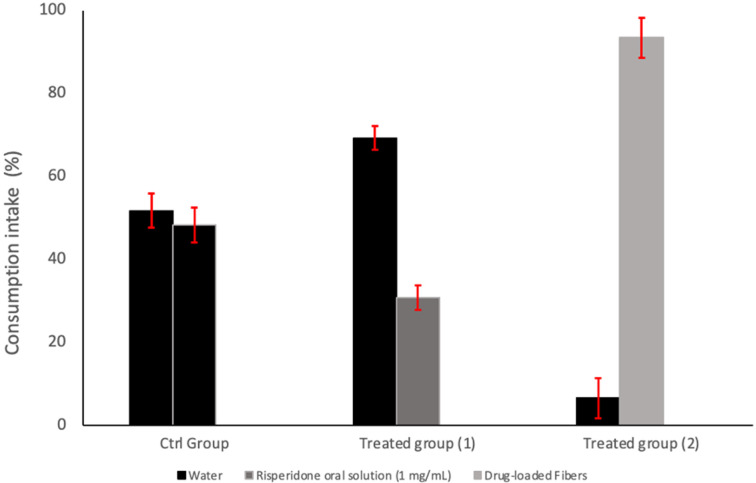
Two-bottle preference test in BALB/c mice. The control group (Ctrl Group) had access to two water bottles. Treated group (1) had access to one bottle of water and one bottle containing commercially available Risperidone oral solution (1 mg/mL). Treated group (2) had access to one water bottle and one bottle containing the dissolved drug-loaded microfibers (1 mg/mL).

The two-bottle preference test demonstrated apparent differences in consumption behavior among the tested groups as illustrated in Figure 8. In the control group, mice consumed nearly equal volumes from both bottles (51% vs. 49%), indicating no significant side bias and confirming the validity of the preference setup. When one of the bottles contained Risperidone oral solution (1 mg/mL) in the Water-risperidone group, a reduction in consumption was observed, with mice favoring plain water (69%) over the risperidone oral solution (31%), consistent with the observations of [63]. In contrast, the Water–Fiber group, which received the drug via a sucrose microfiber solution, exhibited a significant preference for the drug-loaded fiber solution (93%) compared to plain water (7%). This substantial increase in consumption suggests a preference for sucrose fiber loaded with risperidone over water, likely due to the masking effect of sucrose that effectively overcomes the bitter taste of risperidone. The use of sweeteners such as sucrose has been widely highlighted in the literature as an effective strategy to enhance palatability and drug acceptance in rodents [64,65,66]. Furthermore, incorporating bitter drugs into polymer-based delivery systems, such as electrospun and centrifugal-spun fibers, has shown promise in taste-masking and improving patient compliance [67,68,69]. These results support our hypothesis that fabricating bitter taste drugs with intense natural sweeteners such as sucrose can enhance palatability and improve patients’ adherence to medications in pediatric patients, especially those suffering from any sensory challenges, such as ASD. The rejection of the commercially available risperidone oral solution may be attributed mainly to its strong bitter taste, as documented in research highlighting humans’ aversion to risperidone consumption [70]. On the other hand, when given free access to drink from both risperidone oral solution and sucrose microfibers loaded with risperidone, the mice preferred the latter option. It is worth noting that mice rejecting risperidone oral solution is not necessarily due to its bitter taste, but instead to the natural rodent’s avoidance behavior towards any unfamiliar substance introduced to them, or sometimes the avoidance of any substance with an unfamiliar smell [71]. Therefore, the reduced intake might indicate a more general negative response to the drug solution rather than bitterness alone, as is documented [72].

## 4. Conclusions

This study aimed to address the challenges faced by children diagnosed with ASD in taking their medication. Children with ASD often struggle with swallowing pills, leading to delayed treatment and potential health issues. The focus of this study was on risperidone, an antipsychotic drug commonly used to reduce some ASD symptoms. The proposed solution involved the development of rapidly dissolving sugary, drug-loaded fibers that provide a palatable alternative to bitter medicine intake. The fibers exhibited a uniform morphology, rapid disintegration (within 3 s), and successful drug dispersion. The XRD analysis confirmed the absence of any crystalline structures in the drug-loaded fibers, indicating the molecular transformation of risperidone into a solid dispersion due to the centrifugal spinning process. Despite facing some challenges in terms of the EE% and release profile, this formulation represents a promising solution to avoid medicine rejection among ASD children. The drug release assessments indicated an initial burst release followed by a plateau at around 60% after 360 min, which requires further investigation. The in vitro cytotoxicity assessments of risperidone highlighted its safety profile at concentrations ≤ 125 µg/mL, presenting a relatively lower level of toxicity for this antipsychotic drug. The in vivo palatability test revealed that mice preferred the drug-loaded microfibers over the control (water only) and the commercially available risperidone oral solution. In summary, the developed fast-dissolving sugar-based risperidone-loaded microfibers represent a potential solution to enhance medication acceptance among ASD individuals, indicating safety and efficacy. Further studies should focus on improving the EE%, understanding the drug release rate at 60%, and exploring formulation stability under various storage conditions to scale up this technology for practical therapeutic applications. Additionally, pharmacokinetic and pharmacodynamic evaluations are required to confirm the clinical relevance of this formulation.

## 5. Patent

This work was submitted to the Saudi Authority for Intellectual Property as a patent application, currently pending, under submission number 1020253185, dated 7 May 2025.

## Figures and Tables

**Table 1:** Summary of XRD and FTIR results for raw materials, i.e., Sucrose and Risperidone.

Sample	XRD 2θ°	XRD Crystalline Structure	FTIR Wavenumber cm^−1^	FTIR Functional Groups	Refs.
Sucrose	12.49°, 13.51°, 17.49°, 19.05°, 20.44°, 23.01°, 25.48°, 26°, 28.27°, 31.4°, 32.76°, 36.39°, 36.98°, 39.19°, 40.75°, 48.97°.	Crystalline	3500 to 3000 2942–2890 1114 1000 to 700	O-H stretching C-H stretching C-O stretching Carbohydrates C-C	[43,44,45]
Risperidone	7.13°, 10.69°, 11.51°, 14.32°, 14.92°, 15.56°, 16.48°, 18.64°, 19.01°, 19.9°, 21.31°, 22.42°, 23.34°, 23.64°, 27.62°, 28.66°, 29.07°,	Crystalline	3061 2939–2803 1645 1533 1351 1507 994–610 1192 1130	Aromatic C-H stretching C-H stretching Lactam C=O stretching N-H bending Oxazole C-N stretching Aromatic C-C stretching Aromatic C-H bending Tertiary amine C-N stretching Aryl fluoride	[46,47,48]

## Data Availability

The original contributions presented in the study are included in the article; further inquiries can be directed to the corresponding authors.
