# Correction: Alqahtani et al. The Development of Risperidone-Loaded Microfibers via Centrifugal Spinning to Enhance the Palatability of a Potential Drug for Autistic Children. *Pharmaceutics* 2025, *17*, 1403

**DOI:** 10.3390/pharmaceutics18040488

**Published:** 2026-04-16

**Authors:** Sarah H. Alqahtani, Alhassan H. Aodah, Yasser A. Alshawakir, Bayan Y. Alshehri, Ali A. Alamer, Haya A. Alfassam, Fahad A. Almughem, Abdullah A. Alshehri, Essam A. Tawfik

**Affiliations:** 1 Advanced Diagnostics and Therapeutics Institute, Health Sector, King Abdulaziz City for Science and Technology (KACST), Riyadh 11442, Saudi Arabiaaaodah@kacst.gov.sa (A.H.A.); balshehri@kacst.gov.sa (B.Y.A.);; 2 Department of Experimental Surgery and Animal Laboratory, College of Medicine, King Saud University, Riyadh 12372, Saudi Arabia

**Text Correction**

There was an error in the original publication [1]. The patent associated with this research has not been included in the manuscript.

The following section has been added, consisting of one paragraph, to clarify this point:

5. Patent

This work was submitted to the Saudi Authority for Intellectual Property as a patent application, currently pending, under submission number 1020253185, dated 7 May 2025.

The authors state that the scientific conclusions are unaffected. This correction was approved by the Academic Editor. The original publication has also been updated.
